# Non‐*APOE* variants predominately expressed in smooth muscle cells contribute to the influence of Alzheimer's disease genetic risk on white matter hyperintensities

**DOI:** 10.1002/alz.14455

**Published:** 2024-12-31

**Authors:** Hannah Louise Chandler, Joshua Wheeler, Valentina Escott‐Price, Kevin Murphy, Thomas Matthew Lancaster

**Affiliations:** ^1^ School of Physics and Astronomy Cardiff University Brain Research Imaging Centre (CUBRIC) Cardiff University Cardiff UK; ^2^ School of Clinical Sciences University of Bristol Bristol UK; ^3^ Department of Psychology University of Bath Bath UK; ^4^ Centre for Neuropsychiatric Genetics and Genomics Department of Psychological Medicine and Clinical Neurosciences Cardiff University Cardiff UK

**Keywords:** cerebrovascular, polygenic risk score, preclinical, smooth muscles cells, white matter hyperintensities

## Abstract

**INTRODUCTION:**

White matter hyperintensity volumes (WMHVs) are disproportionally prevalent in individuals with Alzheimer's disease (AD), potentially reflecting neurovascular injury. We quantify the association between AD polygenic risk score (AD‐PRS) and WMHV, exploring single‐nucleotide polymorphisms (SNPs) that are proximal to genes overexpressed in cerebrovascular cell species.

**METHODS:**

In a UK‐Biobank sub‐sample (mean age = 64, range = 45–81 years), we associate WMHV with (1) AD‐PRS estimated via SNPs across the genome (minus apolipoprotein E [*APOE*] locus) and (2) AD‐PRS estimated with SNPs proximal to specific genes that are overexpressed in cerebrovascular cell species.

**RESULTS:**

We observed a positive association between non‐*APOE*‐AD‐PRS and WMHVs. We further demonstrate an association between WMHVs and AD‐PRS constructed with SNPs that are proximal to genes over‐represented in smooth muscles cells (SMCs; *β* = 0.135, *P*_FWE_ < 0.01) and internally replicated (*P*_DISCOVERY+REPLICATION _< 0.01).

**DISCUSSION:**

Common AD genetic risk could explain physiological processes underlying vascular pathology in AD. SMC function may offer a treatment target to prevent WMHV‐related AD pathophysiology prior to the onset of symptoms.

**Highlights:**

- Alzheimer's disease (AD) risk factors such as apolipoprotein E (*APOE*) ε4, link to increased white matter hyperintensity volume (WMHV).
- WMHVs indicate vascular risk and neurovascular injury in AD.
- The broader genetic link between AD risk and WMHV is not fully understood.
- We quantify AD polygenic risk score (PRS) associations with WMHV, excluding *APOE*.
- AD‐PRS in smooth muscle cells (SMCs) shows a significant association with increased WMHV.

## 1 INTRODUCTION

Alzheimer's disease (AD) has a complex, polygenic component, and our understanding of specific genetic contributions are limited by heterogeneity in clinical and neurobiological features.^1^ However, recent advances the provide potential for biological specificity including determining how genetic profiles for specific brain cell types are implicated in AD.^2^, ^3^ Cerebrovascular pathophysiology in AD is evident decades prior to symptom onset and is one of the earliest changes to occur in AD development.^4^, ^5^, ^6^ Recently described vascular gene sets are therefore an ideal target for assessing AD‐related pathophysiology with known neurovascular dysfunction.^3^ White matter hyperintensities (WMHs) represent lesions on fluid‐attenuated inversion recovery (FLAIR) magnetic resonance imaging (MRI) scans,^7^ reflecting pathological events including vascular changes, increased blood–brain barrier (BBB) permeability, and myelin degeneration,^8^ and are more severe in individuals with AD.^9^ Although WMH load has been linked to mid‐life AD risk factors/morbidity such as age, hypertension, diabetes, and hyperlipidemia,^10^, ^11^, ^12^ the precise neural processes that lead to the development of white matter hyperintensity volume (WMHV) as a preclinical feature of AD are less established. Increases in WMHV may be independent of established AD pathology and present in asymptomatic individuals years before the onset of clinical symptoms.^13^ Although they frequently co‐occur with other preclinical AD features such as amyloid deposition and cortical thinning, suggestive of a shared contribution to AD development,^14^ the precise mechanism linking increased risk for AD to WMHV load is largely unknown. The association between AD and WMHV may be partly explained by AD genetic risk, such as the association with single‐nucleotide polymorphisms (SNPs) in the apolipoprotein E (*APOE*) locus, where an *APOE* ε4 allele is associated with higher WMH load and increased risk for AD. For example, three independent studies have identified a positive association between *APOE* ε4 status and larger WMHV in UK Biobank.^15^, ^16^, ^17^ However inter‐individual WMHV variation is likely to be genetically complex and multifaceted, with some initial evidence that a non‐*APOE* association between AD polygenic risk score (AD‐PRS) is linked to WMH features via pathways such as cell migration and clearance.^18^ However, little is known about molecular features of the genetic architecture implicated in the association between common AD risk and WMHV. WMHV has a polygenic common genetic architecture, explaining a proportion of an individual's susceptibility to WMHV burden^19^; however, the genetics of AD that confer development of WMHV above and beyond *APOE* and rare missense variations such as *NOTCH3/TRIM3* ^20^, ^21^, ^22^, ^23^ are not well established. PRSs for AD can explain variation across a range of prodromal AD features across the entire lifespan,^2^, ^24^, ^25^, ^26^, ^27^, ^28^, ^29^ before the onset of symptoms.^30^ For instance, we have demonstrated previously that AD‐PRS can shape feature of brain vasculature such as cerebral blood flow^4^, ^5^ decades before the onset of symptoms. Prior studies have demonstrated AD‐PRS is associated with an increased burden of WMHV^31^ in an asymptomatic sample, positioning WMHV as neurobiological antecedent to AD,^32^ and warranting the further investigation we propose here. Understanding of specific biological processes linking AD genetic risk to WMHV could further help clarify individual risk and inform treatment strategies and early intervention/prevention of WMHV. Here, we construct cell‐specific AD‐PRS via genetic variants proximal to genes that are overexpressed in specific vascular cell species, as well as AD‐PRS constructed using established cell types.^3^, ^33^ Specific vascular cell types preferentially harbour AD risk loci, more so than all other brain cell types, apart from established overexpression in microglia. We anticipate that by parsing the AD‐PRS by gene sets linked to specific vascular cell types, we will be able to establish specific biological process that link AD genetic risk to neurovascular injury. This analysis will provide further clarification into the pathophysiology underlying AD‐related alterations in brain health with a specific focus on neurovascular insult.^34^ Understanding preclinical risk factors that confer risk to AD via neuroimaging can help uncover pathophysiological mechanisms that occur decades before the onset of symptoms.

## 2 METHODS

### 2.1 Sample characteristics

Genome‐wide association study (GWAS) summary data were derived from a neuroimaging‐genetic sample of 39,691 individuals from the UK Biobank cohort. Briefly, just under 500,000 individual samples included in the Spring 2018 release of the UK Biobank were first considered. Following genotyping quality‐control procedures for sample removal, a maximally unrelated samples with recent UK ancestry resulted in a subset of 34,298 samples with acceptable genotyping and imaging quality control. Consistent with prior UK Biobank genetic‐imaging approaches, the imaging‐GWAS sample was further randomly divided into sex‐ and age‐matched discovery (*N* = 22,138) and replication cohorts (*N* = 11,086).^35^

### 2.2 WMH imaging

WMHVs were calculated independently via central UK Biobank image‐derived phenotype (IDP) processing, using prior training data established as part of the broader UK Biobank IDP pipeline.^35^ Briefly, WMHV as an IDP was derived using a combination of structural T1‐weighted (three‐dimensional magnetization‐prepared rapid gradient echo [3D MPRAGE], sagittal, *R* = 2, T1/TR 880/2000 ms) and T2‐weighted FLAIR imaging (1.05 × 1.0 × 1.0 mm 192 × 256 × 256, FLAIR, 3D SPACE, sagittal, *R* = 2, PF 7/8, fat sat, T1/TR 1800/5000 ms), and estimated using BIANCA.^36^

### 2.3 WMHV GWAS summary statistic data

The WMHV GWAS summary statistic data were downloaded from UK Biobank (IDP IDs: 1437, IDP_T2_FLAIR_BIANCA_WMH_volume), available at https://open.win.ox.ac.uk/ukbiobank/big40/release2. After removing individuals with no available genetic data (*N* = 2184), the imaging‐genetic data set comprised 32,114 (*N*_DISCOVERY_ = 21381; *N*_REPLICATION_ = 10,733) participants, which was corrected for an extensive array of confounds (including subject specific, such as sex, age, head size, motion; scanner specific; table position; and genetic ancestry and relatedness) as employed previously to optimize confound modeling in neuroimaging UK Biobank data.^35^, ^37^ The WMHV GWAS summary statistic data were considered as our exposure/outcome, where SNPs/beta coefficients are aligned to the AD‐PRS exposure instruments (see Section 2.6 for further clarification).

### 2.4 AD‐PRS generation

In this study, all SNPs with a low minor allele frequency (MAF <0.01) and lower imputation quality (INFO <0.9) were excluded. AD‐PRS were created using PRSice v1.25 risk profile software^38^ using a stringent clumping procedure [clump.kb = 10mb, clump.r2 = 0.001] to remove correlated alleles, with 1000 Genomes Project (phase 3) as reference data to model linkage disequilibrium. To estimate whole‐genome AD‐PRS and cell‐type specific derivatives, we included SNPs across a range of *p*—thresholds at *p* < 0.01–1.0 from the a recent AD‐GWAS described in Kunkle et al.,^39^ with minimal known inclusion or overlap with individuals from UK Biobank.^40^ For the whole‐genome non‐*APOE* AD‐PRS, all SNPs across the genome were considered (regardless of SNP–gene annotation), apart from SNPs within the *APOE* (chr 19: 44,500–46,000 kb) and the major histocompatibility complex (MHC) (chr 6: 26,000–34,000 kb) regions, which were removed from the pruned data to ensure putative associations where not influenced by prior *APOE*‐linked observations or bias from complex linkage disequilibrium (LD) structure.^41^, ^42^, ^43^ As a sensitivity analysis, we added the *APOE* locus back to the AD‐PRS model to establish if *APOE* effects were consistent with prior observations in the UK Biobank.^15^, ^44^

**RESEARCH IN CONTEXT**

1. **Systematic review**: We reviewed the literature linking white matter hyperintensity (WMH) with Alzheimer's disease (AD) and genetic risk factors that were considered previously such as apolipoprotein E (*APOE*) *ε4*. We assessed studies exploring polygenic risk score (PRS) and its association with WMH volume (WMHV) and current models that increase biological interpretation such as cell‐specific AD‐PRS.
2. **Interpretation**: Our findings first show a non‐specific, whole‐genome positive association between non‐*APOE* AD‐PRS and WMHV. We further identified a link between WMHV and AD‐PRS in smooth muscle cells (SMCs), suggesting a specific genetic basis for vascular pathology in AD. Common AD risk alleles proximal to genes overexpressed in SMCs increases WMHV load before the onset of AD.
3. **Future directions**: Future research should investigate the role of SMC function in AD‐related WMHV. Studies should focus on how these genetic factors influence vascular health and explore potential treatments targeting SMC to prevent WMHV‐related pathophysiology before AD symptoms appear.

### 2.5 Cell‐type specific AD‐PRS

After AD‐PRS quality control, pruning, and estimation, *N* = 23,869 uncorrelated SNPs were brought forward for consideration. We further mapped these SNPs to proximal genes using “g:SNPense” function provided as part of the gprofiler2 toolbox.^45^ Briefly, “g:SNPense” maps a list of human SNP rs‐codes to corresponding gene names, retrieves chromosomal coordinates, and predicts variant effects. The mapping process is limited to variants that overlap with at least one protein‐coding gene. The “gSNPense” function retrieves genome variant SNP data from dbSNP, mapping to specific genes from National Center for Biotechnology Information (NCBI) gene database,^46^ using upstream/downstream cutoffs of 2 and 0.5 kbps, respectively.^47^ After removing any SNPs that were mapped to the same gene in multiple instances, a total of 754 SNPs were considered for AD‐PRS estimation. We further considered AD‐PRS for specific genes that are preferentially expressed in specific cell types across the vasculature and other brain cell types, as identified by Yang et al.^48^ We considered AD‐PRS consisting of SNPs mapped to genes that were disproportionally expressed across all 14 brain cell types, including a series of recently described vascular cell species such as endothelial, pericyte, ependymal, and smooth muscle cell (SMC) species (see Tables S1–S3, for all (1) cell types considered and (2) the number of SNPs mapped to each specific cell type AD‐PRS). We define genes “overexpressed” as those serving as specific markers/show enriched expression in particular cell types as outlined in Yang et al.^48^ This enrichment is measured as a log‐transformed fold change, comparing expression levels in each cell type to background levels. Significance is assessed using cumulative hypergeometric testing, corrected for the false discovery rate. To first assess the validity/reliability of this cell‐specific AD‐PRS model approach, we first perform a pseudo‐replication of Yang et al.,^33^ who assessed the relationship between cell‐specific AD‐PRS and AD diagnosis. Here, we explore a whole‐genome AD‐PRS and six cell‐specific AD‐PRS, using SNP effect sizes from the Kunkle et al.^39^ AD GWAS summary statistics as our exposure/independent variable instruments and SNP effect sizes from the UK Biobank Family history/AD‐by‐proxy GWAS^49^ as our outcome. Here we observed a ranked set of cell‐specific AD‐PRS effect sizes comparable to Yang et al.,^33^ where whole genome AD‐PRS explained the most variance in AD as an outcome in our analysis (*rho* = 0.82, *p* = 0.034; see Figure S1).

### 2.6 Statistical analysis

We employ a PRS approach using the “gtx” method first described by Johnson^50^ for regressing the response variable onto the risk score first described in previous studies.^2^, ^51^, ^52^ This PRS approach is equivalent to the “inverse variance weighted” (IVW) approach in two‐sample Mendelian randomization studies. However, in PRS analysis, there is no stringent inclusion criteria for genetic variants: we do not require the variants to be strongly associated with the exposure and pleiotropic effects are allowed. Briefly, the risk score coefficients, represent the “weights” used to compute the risk score for a set of SNPs (in this case, the exposure—AD‐PRS), measured in units per dose of the coded allele. Typically, these weights are single‐SNP regression coefficients estimated from AD GWAS summary statistic data.^39^ The aligned beta coefficients reflect the regression coefficients for the outcome variable (in this case, the outcome—WMHV GWAS summary statistics), calculated for the same set of SNPs and using the same coded allele as for weights, typically estimated from single‐SNP regression models, but from an independent GWAS summary statistic data set. In causal inference studies, the aim is often to estimate the causal effect of an intermediate trait or biomarker on an outcome variable. Here, weights represent the estimated effects on the intermediate trait or biomarker, whereas the beta coefficient captures the estimated effects on the outcome variable and its standard errors. The sample size, *n*, is used to compute the (pseudo) variance explained in the testing data set, derived from the likelihood ratio test statistic. This method is exact when SNPs are uncorrelated (in this case, *R* ^2^ < 0.001) and when a quadratic log‐likelihood is used, which can be obtained under a normal linear model or any regression model with a large sample size. We employ a family‐wise error (FWE) rate alpha to the beta/*p*‐values independently considering all 14 cell‐specific AD‐PRS and 3 progressive *p*‐thresholds. In order to ascertain whether a significant cell‐specific AD‐PRS SNP set size was contributing to putative explained variance, the relationship between AD‐linked WMHV was validated using a permutation analysis that was conducted to set an empirical threshold by creating AD‐PRS from 1000 permuted SNP sets,^53^ controlling for SNP set size and *p*‐threshold. To assess the generalizability of specific cell‐specific AD‐PRS sets associated with WMHV, we performed a split‐sample approach, where we employ GWAS from two samples of 21,360 and 10,727.

## 3 RESULTS

### 3.1 Whole‐genome and cell‐specific AD‐PRS associations with WMH‐V

**FIGURE 1:**
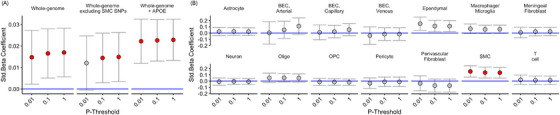
(A) Whole‐genome AD‐PRS; minus the SMC SNPs and including the *APOE* region. (B) Cell type–specific AD‐PRS. Beta coefficients in red reflect *p*_FWE_‐CORRECTED < 0.05). Error bars reflect 95% confidence intervals of the beta coefficient for the combined sample (*N* = 32,087). *APOE*, apolipoprotein E; BEC, brain endothelial cell; FWE, family‐wise error; SMC, smooth muscle cell.

**FIGURE 2:**
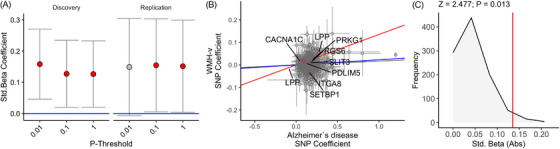
(A) Association between AD‐PRS and WMHV. Red beta coefficients reflect *p*_FWE_‐CORRECTED < 0.05. Error bars reflect 95% confidence intervals of the beta coefficient for discovery (*N* = 21,360) and replication samples (*N* = 10,727). (B) The association between SNP effect sizes for AD and WMHV for the whole‐genome AD‐PRS (gray line‐of‐best‐fit), a whole‐genome AD‐PRS (including *APOE*—blue line‐of‐best‐fit), and SMC AD‐PRS (red line‐of‐best‐fit). Gene IDs reflect gene proximal to SNPs in the SMC AD‐PRS. Each SNP is plotted by coefficient in the AD risk (*x* axis) versus estimated effect size for WMHV in the independent data set (*y* axis). (C) The SMC AD‐PRS was associated with WMHV to a higher degree compared to the majority of a 1000 SNP‐set size‐matched, randomly sampled AD‐PRS (two tailed). Vertical red line represents degree of association (absolute standard beta coefficient) between SMC AD‐PRS and WMHV. FWE, family‐wise error; SMC, smooth muscle cell; WMHV, white matter hyperintensity volume.

We observed a positive association between whole‐genome AD‐PRS and WMHV, across all AD‐PRS *p*‐thresholds (*β*_LOWEST_ > 0.01, *p*_FWE_ < 0.05, Figure 1A—whole‐genome). We then separated the AD‐PRS into an extended set of 14 cell‐specific AD‐PRS and repeated the analyses. We observed that the SMC AD‐PRS was positively associated with WMHV (*β* > 0.13, *p*_FWE_ < 0.05, Figures 1B and 2B). (See Table S1 for all associations.) The SNPs within the SMC AD‐PRS did not fully explain the full‐genome AD‐PRS association, as removing the SNPs from the full‐genome AD‐PRS analysis remained significant (*p* < 0.03, in all cases) and was not significantly attenuated (Figure 1A—whole‐genome excluding SMC SNPs). Consistent with prior positive associations between *APOE* and WMHV, we further observed that including the *APOE* locus in the AD‐PRS model increased the strength of the association (*β* = 0.025 ± 0.0053, *p* = 1.49e‐06 Figure 1A—whole‐genome + *APOE*). In order to assess the generalizability of this finding, we demonstrate that the positive SMC AD‐PRS–WMHV association could be observed in both the discovery (*N* = 21,360; *β*_LOWEST_ > 0.12, *p*_HIGHEST_ < 0.010) and replication (*N* = 10,727; *β*_LOWEST_ > 0.14, *p*_HIGHEST _< 0.031) samples (see Figure 2A, Tables S2 and S3 for all associations). We further demonstrate that the association between SMC AD‐PRS and WMHV was not linked to SNP set size, as AD‐PRS constructed of random AD risk SNPs of comparable set sizes did not associate with WMHV in a comparable manner (*Z* = 2.47; *p* = 0.013, 1000 simulations—Figure 2C).

## 4 DISCUSSION

Here we investigated how gene sets expressed within a range of cell types (including a novel array of genes expressed more in specific vascular cell species^3^) influence AD by contributing to WMHV burden. Our initial analysis revealed a positive association between whole‐genome AD‐PRS and WMHV for all AD‐PRS *p*‐thresholds. When constructing AD‐PRS from genes preferentially expressed in 14 cell‐specific subtypes, we observed a significant association between the genes that are expressed in vascular SMCs and WMHV. Although our observations corroborate prior accounts linking *APOE* loci to WMHV in the UK Biobank,^15^, ^16^, ^17^ all of which suggest that AD risk at the *APOE* locus increases WMHV, this is the first evidence to our knowledge linking AD genetic risk in SMCs to WHMV and to any AD imaging biomarkers, more broadly. We also note that we observed a similar, but weaker (and not significant after multiple testing) association between WMHV and AD‐PRS restricted to genes expressed in ependymal cells, which line the ventricles proximal to typical sites of periventricular WMHV presentation,^54^ adding construct validity to our inferences.

In the brain, SMCs surround arteries and arterioles, moderating key functions in the neurovascular unit and regulating cerebral blood flow to meet tissue energy demands.^55^ Increased WMHV burden is associated with impaired cerebrovascular reactivity (CVR) and reduced vasoconstriction,^56^, ^57^ indicating a dysfunction in SMCs. In healthy white matter, impaired CVR also precedes the development of WMHV, suggesting that dysfunction in contractability may contribute to the pathogenesis of cerebral small vessel disease and the development of WMH burden.^58^ SMCs are also implicated in the early development and progression of atherosclerosis,^59^, ^60^, ^61^ a condition highly prevalent in AD^62^ and also associated with the development of WMH burden.^63^ Furthermore, SMCs regulate blood pressure by altering the luminal diameter to constrict and relax blood vessels.^64^ In hypertensive conditions, chronic vasoconstriction or vasodilation/reduced compliance may lead to reduced blood flow, oxygen deprivation, and hypoxia contributing to microvascular damage and WMH burden.^65^ However, the role of SMCs in WMH development is multifaceted and not fully understood. The precise causal process, as well as moderating effects of environmental risk factors, remain the subject of ongoing investigation.

Recent evidence demonstrates associations between WMHV and AD, even when accounting for genetic confounding from pulse pressure,^32^ suggesting that targeting vascular pathophysiology and WMHs is an important to consider for intervention/prevention strategies. Despite identifying a specific vascular link between WMHs and AD genetic risk, the pathogenesis and genetic basis of WMHs is still poorly understood. As a model mechanism of action, one of the pathophysiological markers of Cerebral Autosomal Dominant Arteriopathy with Subcortical Infarcts and Leukoencephalopathy (CADASIL) and mutations in the *NOTCH3* gene includes an increase in WMHs and a loss of vascular smooth muscle cells.^66^ The pathophysiological correlates of WMHs in CADASIL suggest lower baseline total cerebral blood flow in *NOTCH3* mutation carriers that precedes the development of WMHs.^67^ This may suggest that changes in the mechanisms that help regulate cerebral blood flow (including SMCs) and reduce pulsatile pressure can lead to downstream microvascular damage resulting in the development of WMHs.

SMCs are expressed in both superficial (sub‐cortical arteries)^55^ and deep (medullary arteries) white matter vasculature before branching into arterioles and capillaries.^68^ These arteries (and the associated veins) are surrounded by perivascular spaces (PVSs), which form part of the glymphatic system and are important conduits of brain drainage implicated in neurovascular injury.^69^, ^70^, ^71^ Interstitial fluid and solutes diffuse through extracellular spaces at the capillary level and then drain between the capillary endothelial basement membrane and the SMC basement membrane.^72^, ^73^ Pathophysiological events in this process lead to BBB breakdown and microbleeds such as cerebral amyloid angiopathy (CAA), where amyloid beta is deposited into the basement membranes of SMCs^74^ and in CADASIL, where loss of arterial SMCs reduces the amplitude of pulsations that facilitate the drainage along PVSs. Vascular SMCs carrying the mutated *NOTCH3* receptor diminish, thereby reducing SMC function, leading to abnormal arterial tone and contractibility.^75^ This cerebrovascular dysfunction often precedes WMHV increases.^76^ Both CAA and CADASIL are associated with microbleeds and increased WMHV. Examination of white matter in these conditions reveals that vascular degeneration progresses from a loss of SMCs.^77^ Although other cerebrovascular cell species, such as pericytes^78^ and endothelial cells,^79^ have been linked to AD through BBB dysfunction, we did not observe any evidence of common AD genetic risk driving an association with WMHV via SNPs linked to genes preferentially expressed in these cell types. If changes in pericyte or endothelial function are associated with AD‐PRS and are present in a largely asymptomatic group, T2‐FLAIR MRI may not have the sensitivity to assess dysfunction at the capillary/BBB level. However, SMC dysfunction because of increased SMC AD‐PRS burden could lead to macrostructural alterations such as elevated WMHV and other observable presentations such as microbleeds.

We suggest that these observations are considered with the following limitations. First, we acknowledge that the impact of both genome‐wide and SMC AD‐RPS on WMHV is minimal, explaining less than 0.05% of the variance in both cases. Second, because WMHV was collected in mid‐ to later‐life, we cannot determine whether these associations are fixed or dynamic throughout the lifespan. Third, although we acknowledge that the *p*‐thresholds we use have good predictive performance, we cannot fully infer the potential causal inference between genetic risk for AD and WMHV, and our associations may be biased by horizontal pleiotropy. Given these collective limitations, we advise caution when interpreting the effect sizes and variance explained by whole‐genome and SMC AD‐RPS in this study. Fourth, we only considered SNPs proximal to genes for brain cell–specific species, not biological function or subtype or cell state. We also suggest that fine‐mapping SNPs with known downstream function may also improve the performance in such pathway‐specific approaches.^80^, ^81^ Future bioinformatics research should aim to refine and uncover the principal biological gradients that underpin AD genetic risk.^82^ This research will help delineate the various AD‐linked processes that may contribute to WMHV across both early and later life stages.

To conclude, we demonstrate that there is a common genetic risk factor for AD associated with an increased WMHV burden expressed in SNPs that are proximal to genes disproportionally expressed in SMCs. Further experimental models are required to establish the precise mechanisms and to explore potential therapeutic strategies to target SMC action in WMHV‐related AD morbidity. Understanding the links between AD genetic risk and WMH holds promise for advancing our comprehension of AD and may provide implications for early diagnosis, personalized risk assessment, and the development of targeted therapeutic interventions. Understanding the role of SMC in the development of AD could therefore be instrumental in the development toward interventions to prevent or mitigate WMH burden and attenuate associated cognitive impairment and other neurodegenerative sequalae.

## CONFLICT OF INTEREST STATEMENT

All authors declare that they have no competing interests. Author disclosures are available in the supporting information.

## CONSENT STATEMENT

All participants were recruited to the wider UK Biobank study, and provided informed consent. UK Biobank has approvals from the Northwest Multi‐centre Research Ethics Committee (MREC) as a Research Tissue Bank (RTB). This approval means that researchers do not require separate ethical clearance and can operate under the RTB approval (there are certain exceptions to this which are set out in the Access Procedures, such as re‐contact applications). https://www.ukbiobank.ac.uk/media/p4yjfqcp/2021‐nwrec‐rtb‐application‐and‐approval.pdf.

## Supporting information



Supporting Information

Supporting Information

Supporting Information
