# Supplementary material for: Non‐APOE variants predominately expressed in smooth muscle cells contribute to the influence of Alzheimer's disease genetic risk on white matter hyperintensities
Source: Alzheimers Dement. 2024 Dec 31;21(2):e14455. doi: 10.1002/alz.14455 (PMC11848156; doi:10.1002/alz.14455)
Supplement: Supplementary file 1 — Supporting Information [file ALZ-21-e14455-s002.docx]

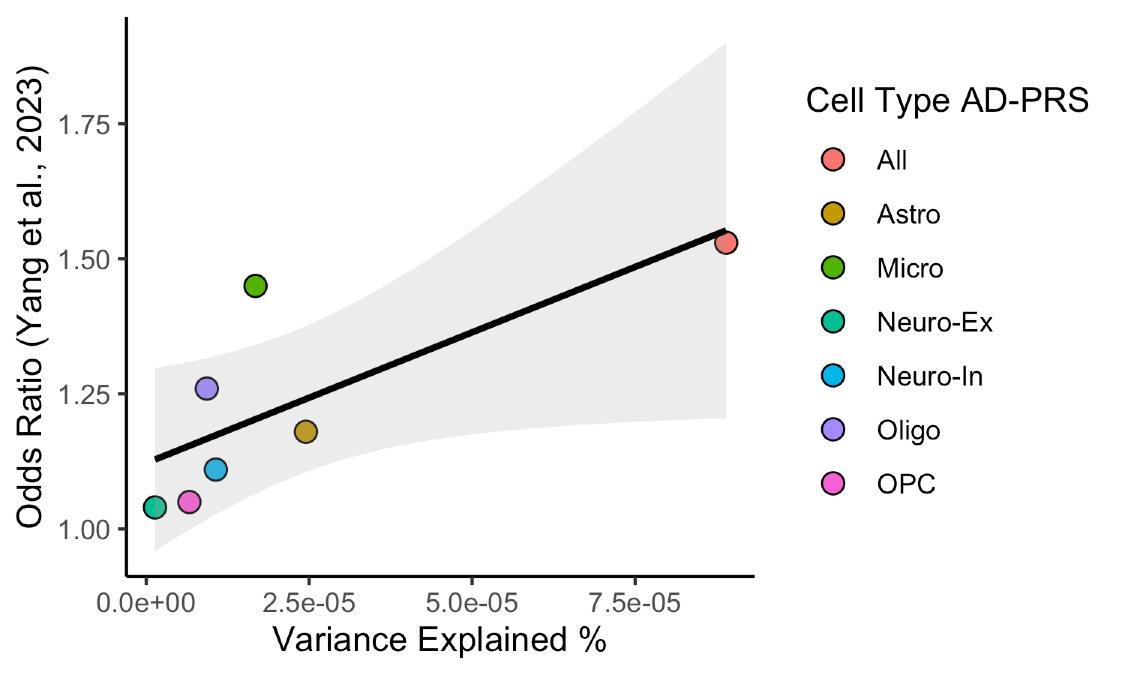
Supplementary Figure 1. Scatter plot depicts y-axis: the effect sizes for the association between AD-PRS and AD diagnosis across the whole genome and for cell-type specific AD-PRS – taken from Figure 2a. in Yang et al. 2023 (33); x-axis effect sizes for AD-PRS and AD-diagnosis-by proxy (maternal / paternal AD diagnosis) for whole genome and for cell-type specific AD-PRS using current methodology.
